# Identification of Shigatoxigenic and Enteropathogenic *Escherichia coli* Serotypes in Healthy Young Dairy Calves in Belgium by Recto-Anal Mucosal Swabbing

**DOI:** 10.3390/vetsci7040167

**Published:** 2020-10-31

**Authors:** Audrey Habets, Frederik Engelen, Jean-Noël Duprez, Brecht Devleesschauwer, Marc Heyndrickx, Lieven De Zutter, Damien Thiry, Eric Cox, Jacques Mainil

**Affiliations:** 1 Laboratory of Bacteriology, Department of Infectious Diseases, Institute for Fundamental and Applied Research in Animals and Health (FARAH) and Faculty of Veterinary Medicine, University of Liège, Quartier Vallée II, Avenue de Cureghem 6, B-4000 Liège, Belgium; Audrey.Habets@uliege.be (A.H.); Jean-Noel.Duprez@uliege.be (J.-N.D.); jg.mainil@uliege.be (J.M.); 2 Laboratory of Immunology, Department of Virology, Parasitology and Immunology, Faculty of Veterinary Medicine, Ghent University, Salisburylaan 133, B-9820 Merelbeke, Belgium; Frederik.Engelen@UGent.be (F.E.); eric.cox@ugent.be (E.C.); 3 Department of Epidemiology and Public Health, Sciensano, Rue Juliette Wytsmanstraat 14, B-1050 Brussels, Belgium; brecht.devleesschauwer@sciensano.be; 4 Department of Veterinary Public Health and Food Safety, Faculty of Veterinary Medicine, Ghent University, Salisburylaan 133, B-9820 Merelbeke, Belgium; lieven.dezutter@ugent.be; 5 Institute for Agricultural and Fisheries Research, Unit Technology and Food, Brusselsesteenweg 370, B-9090 Melle, Belgium; marc.heyndrickx@ilvo.vlaanderen.be; 6 Department of Pathology, Bacteriology and Poultry Diseases, Faculty of Veterinary Medicine, Ghent University, Salisburylaan 133, B-9820 Merelbeke, Belgium

**Keywords:** *Escherichia coli*, EPEC, STEC, dairy calves, recto-anal mucosal swab, Belgium

## Abstract

Enterohemorrhagic *Escherichia coli* (EHEC), enteropathogenic *E. coli* (EPEC), and Shigatoxigenic *E. coli* (STEC) are carried by healthy adult cattle and even more frequently by young calves in their intestinal tract, especially at the height of the recto-anal junction. The purpose of the present study was to assess the presence of ten EHEC, EPEC, and/or STEC O serotypes (O5, O26, O80, O103, O111, O118, O121, O145, O157, and O165) in calves sampled via recto-anal mucosal swabs (RAMS) at three dairy farms in Belgium. A total of 233 RAMS were collected on three consecutive occasions from healthy <6-month-old Holstein-Friesian calves and submitted to a PCR targeting the *eae*, *stx1*, and *stx2* genes after non-selective overnight enrichment growth. The 148 RAMS testing positive were streaked on four (semi-)selective agar media; of the 2146 colonies tested, 294 from 69 RAMS were PCR-confirmed as EHEC, EPEC, or STEC. The most frequent virulotype was *eae*+ EPEC and the second one was *stx1*+ *stx2*+ STEC, while the *eae*+ *stx1*+ and *eae*+ *stx1*+ *stx2*+ virulotypes were the most frequent among EHEC. The majority of EHEC (73%) tested positive for one of the five O serotypes detected (O26, O103, O111, O145, or O157) vs. 23% of EPEC and 45% of STEC. Similarly, more RAMS (73%) harbored EHEC isolates positive for those five serotypes compared to EPEC (53%) or STEC (52%). This survey confirms that (i) healthy young dairy calves are asymptomatic carriers of EHEC and EPEC in Belgium; (ii) the carrier state rates, the virulotypes, and the identified O serotypes differ between farms and in time; and (iii) a majority of EPEC belong to so far unidentified O serotypes.

## 1. Introduction

Enterohemorrhagic *Escherichia coli* (EHEC) are important human pathogens responsible for hemorrhagic colitis (HC), the origin of their name, and the life-threatening hemolytic-uremic syndrome (HUS). Their most important virulence-associated properties are the Shiga toxins (Stx1 and/or Stx2), encoded by phage-located genes (*stx1* and *stx2*), and the production of the histological attaching–effacing (AE) lesion, encoded by genes located on a pathogenicity island (the locus of enterocyte effacement or LEE), including the *eae* gene coding for the intimin adhesin. There exists a broad variety of human EHEC serotypes of whom O26:H11, O103:H2, O111:H-, O121:H19, O145:H-, O157:H7, and O165:H25 (the so-called “gang-of-seven”) are the most frequent and pathogenic worldwide, and O157:H7 and O26:H11 represent the majority of confirmed human cases in the EU [1,2,3,4]. However, not all Stx- and AE-producing *E. coli* cause HC in humans and, therefore, the acronym EHEC is not appropriate for them. Therefore, the acronym AE-STEC, after attaching–effacing- and Shiga-toxin-producing *E. coli*, will be used throughout this manuscript, as previously proposed [5].

Human infection most frequently occurs via the consumption of animal- or plant-derived foodstuffs contaminated with ruminant, mostly cattle, feces. Cattle can indeed be asymptomatic carriers in their intestinal tract, more especially in the colon and at the height of the recto-anal junction [2,6,7,8]. Furthermore, young calves are more frequently healthy carriers of AE-STEC than older animals [9,10,11]. In addition, AE-STEC are also responsible for diarrhea in <3-month-old calves. The majority belong to some of the “gang-of-seven”, such as O26:H11 and O111:H-, and to a few other serotypes, such as O5:H-, O80:H2, and O118:H16 [12,13,14].

Besides AE-STEC, enteropathogenic *E. coli* (EPEC) harbor the LEE and the *eae* gene and produce the AE lesion but no Stx, while STEC sensu stricto produce Stx but no AE lesion. EPEC are responsible for diarrheic diseases in humans and several animals, including young calves. EPEC can belong to host-specific serotypes (such as O127 in humans or O15 in rabbits) or to host-non-specific, including the “gang-of-seven”, serotypes (such as O26:H11, O80:H2, and O111:H- in calves). However, the serotypes of the majority of bovine EPEC remain unidentified. As with AE-STEC, EPEC and STEC are also present in asymptomatic cattle [1,12,13,14,15,16,17].

The purpose of the present study was to assess the presence of the “gang-of-seven” and of three other (O5, O80, and O118) (AE-)STEC and EPEC O serotypes in healthy <6-month-old dairy calves sampled via recto-anal mucosal swabs (RAMS) at three farms [18,19] by following a serotype non-specific enrichment and isolation procedure [17].

## 2. Material and Methods

### 2.1. Farms, Animals, and Sampling

A total of 233 RAMS were collected from <6-month-old Holstein-Friesian calves at 3 Belgian dairy farms situated in East Flanders (74 in FarmA, 71 in FarmB, and 88 in FarmC) on 3 consecutive occasions (65 at Sampling1, 75 at Sampling2, and 93 at Sampling3) 2 to 4 weeks apart between June and October 2018 [20]. During the survey, 18 calves in FarmA, 19 calves in FarmB, and 11 calves in FarmC were sampled twice, while 6 calves in FarmA and 13 calves in FarmC were sampled three times. Sterile cotton prepping balls (Covidien, Dublin, Republic of Ireland) were inserted into the anus of the calves and circular motions were applied to sample the entire mucosal surface of the recto-anal junction. Clinical signs (diarrhea), if any, were recorded at each visit. Fecal sampling by recto-anal swabbing is considered non-invasive. Therefore, the calves in this study did not fall into the definition of experimental animals and no ethical approval was required.

### 2.2. Preliminary Screening

All RAMS were transported on ice to the laboratory of UGent, stored overnight at 4 °C, and homogenized in 25 mL of maximum recovery diluent (Oxoid, Dilbeek, Belgium) by stomaching [20]. At ULiège, the whole procedure to isolate EPEC and (AE-)STEC was slightly adapted from Thiry and collaborators [17]. 

Briefly, 100 µL of the suspension was added to 5 mL of lauryl sulfate broth (Sigma-Aldrich, Darmstadt, Germany) and incubated overnight at 37 °C. Bacterial DNA was extracted from 1.5 mL of the enrichment broths via the alkaline-boiling method and stored at −20 °C. Lysates were tested with a triplex PCR targeting the *eae*, *stx1*, and *stx2* genes [21]. Each PCR-positive broth was subsequently streaked onto four (semi-)selective agar plates and incubated overnight at 37 °C: McConkey’s (MC), Chromocult Coliform ES (ES) (VWR, Leuven, Belgium), Chromocult Coliform ES supplemented with 2.5 mg/mL of potassium tellurite (ESTe) (Sigma-Aldrich, Darmstadt, Germany) [22], and supplemented CHROMagarTM STEC base (STECB) (I2A, Montpellier, France).

### 2.3. Identification of Virulotypes and O Serotypes

Up to 5 colonies per agar plate were picked up, inoculated into 200 µL Luria–Bertani (LB) broth in 96-well microtiter plates, and tested by colony hybridization (CH). CH was performed with PCR-derived 32P radioactively labeled gene probes targeting the *eae*, *stx1*, and *stx2* genes to identify EPEC and (AE-)STEC [23]. After overnight incubation at 37 °C, 1 µL from each well was transferred onto LB agar using a transfer comb, followed by overnight incubation at 37 °C. The colonies were transferred by contact onto Whatman 541 paper filters (VWR, Leuven, Belgium) and treated to lyse the cells and denature the DNA. All probe-positive colonies were stored at −80 °C in LB broth with 40% glycerol until further use.

Probe-positive colonies were subsequently grown overnight on LB agar plates at 37 °C. DNA was extracted from a single colony by the alkaline-boiling method and subjected to the triplex PCR for the *eae*, *stx1*, and *stx2* genes for confirmation of the virulotypes [21,24]. Isolates with CH/PCR discordant results were retested with the PCR. Finally, confirmed triplex PCR-positive colonies were tested by PCR for the ten O serotypes listed above: the “gang-of-seven”, O5, O80, and O118 [21].

## 3. Results

### 3.1. PCR-Positive RAMS

Of the 233 RAMS after overnight enrichment in lauryl sulfate, 148 tested positive with the triplex PCR (Table 1): 49 in FarmA (66%), 47 in FarmB (66%), and 52 in FarmC (59%). The number of PCR-positive RAMS increased with the sampling time (Table 1): 13 RAMS (20%) were PCR-positive at Sampling1 vs. 50 RAMS (77%) at Sampling2 and 85 RAMS (91%) at Sampling3. Eleven of the PCR-positive RAMS were sampled from calves with signs of diarrhea. All 148 PCR-positive broths were subsequently streaked on MC, ES, ESTe, and STECB agar plates and incubated overnight at 37 °C.

### 3.2. CH- and PCR-Positive Colonies

Of the 2146 colonies studied, 721 were isolated on MC, 711 on ES, 233 on ESTe, and 481 on STECB media. Of these, 338 (16%) from 77 RAMS (52%) tested positive with the probes for the *eae*, *stx1*, and/or *stx2* genes (Table 1). Of these 338 CH-positive colonies, 294 (87%) from 69 RAMS (90%) were confirmed by the triplex PCR (Table 1). Seven of the eight RAMS with no confirmed PCR-positive colonies had only one or two CH-positive colonies. Some CH-positive PCR-negative colonies were also observed in 20 other RAMS. The CH and PCR results were in agreement for 257 PCR-positive colonies (87%). 

In summary, 69 of the 148 PCR-positive RAMS (47%) and of the 233 collected RAMS (30%) harbored PCR-positive colonies. Those percentages in the three farms were 31% and 20% (FarmA), 55% and 37% (FarmB), and 54% and 32% (FarmC), respectively. The same respective percentages according to the sampling time were 31% and 6% (Sampling1), 42% and 28% (Sampling2), and 52% and 47% (Sampling3). However, the percentage of triplex PCR-positive RAMS increased differently with sampling in the three farms. In FarmA it increased only at Sampling3 (6%, 4%, 38%), in FarmB it increased at Sampling2 but stabilized at Sampling3 (9%, 46%, 55%), and in FarmC it increased consecutively from Sampling1 to Sampling2 and to Sampling3 (4%, 32%, 51%).

Seventeen RAMS harbored only one PCR-positive colony, while the remaining 52 RAMS harbored up to 15 PCR-positive colonies. Of the 11 PCR-positive RAMS from diarrheic calves, only one PCR-positive colony was isolated in FarmA at Sampling3.

### 3.3. Virulotypes of the PCR-Positive Colonies

The 294 PCR-positive colonies from 69 RAMS were identified as EPEC (131 isolates including the one from one diarrheic calf), AE-STEC (89 isolates), or STEC (74 isolates) (Table 2). EPEC were identified in 36 of the 69 RAMS with PCR-positive colonies (52%), AE-STEC in 23 RAMS (33%), and STEC in 27 RAMS (39%). Of the 52 RAMS with more than one PCR-positive colony, 4 RAMS from FarmA, 6 RAMS from FarmB, and 1 RAMS from FarmC harbored *E. coli* belonging to two different pathotypes, while 3 RAMS from FarmB and 1 RAMS from FarmC harbored *E. coli* belonging to three different pathotypes. 

The most frequent virulotype among colonies was *eae*+ EPEC and the second one was *stx1+ stx2+* STEC. The *eae+ stx1+* and *eae+ stx1+ stx2+* virulotypes were the most frequent among AE-STEC, while the *eae+ stx2+* AE-STEC and the *stx1+* STEC virulotypes were the least frequent (Table 2). 

No calf in FarmA sampled either two or three times was positive more than once, while two calves in FarmB sampled twice were twice positive, three calves in FarmC sampled three times were positive twice (at the second and third samplings), and one calf in FarmC sampled three times was positive at each sampling. The virulotypes were identical (*eae+* and *eae+ stx1+ stx2+*) in only the two calves in FarmB that were sampled twice.

### 3.4. O Serotypes of the PCR-Positive Colonies

Of the 294 PCR-positive colonies, 136 (46%) tested positive for 5 of the 10 serotypes searched for: O26, O103, O111, O145, or O157 (Table 3). The majority of AE-STEC (73%) tested positive for one of the five O serotypes detected vs. a minority of EPEC (23%) and almost half of STEC (45%). Similarly, a majority of RAMS harboring AE-STEC isolates (83%) were positive for one of those five serotypes vs. half of the RAMS harboring EPEC (53%) or STEC (52%) (Table 3). Nevertheless, 65% of the RAMS with EPEC and/or (AE-)STEC harbored isolates not belonging to the “gang-of-seven” serotypes. 

The most frequent were the O111 (62 colonies from 18 RAMS) and O145 (43 colonies from 16 RAMS) serotypes, while the O26, O103, and O157 serotypes were the least frequent. In 27 RAMS, all PCR-positive colonies, including the one from the diarrheic calf, tested negative with the PCR for the 10 searched-for serotypes. The identified serotypes were not equally distributed among the different virulotypes, but they were all identified only among *eae*+ EPEC. Of the 52 RAMS with more than one PCR-positive colony, 34 (65%) harbored EPEC, AE-STEC, and/or STEC belonging to different serotypes, including unidentified ones. 

The O26 serotype was identified only in FarmB, while the O157 and O103 serotypes were not detected in FarmB and FarmC, respectively (Table 4). The number of serotypes identified actually increased with sampling time: from two (O111 and O145) at Sampling1 to four at Sampling2 (O26, O111, O145, and O157) and all five at Sampling3 (O26, O103, O111, O145, and O157). Similarly, the number of RAMS with typeable isolates also increased with sampling time, more especially those positive for the O111 and O145 serotypes (Table 4).

These same two serotypes, O111 and O145, were identified along with other serotypes in the six calves sampled either two or three times in FarmB and FarmC. Nevertheless, only one of the two calves positive twice in FarmB had the same serotype (O111) identified at both samplings (all isolates were *eae+ stx1+ stx2+*), and the calf positive three times in FarmC had the same serotype (O111) identified at Sampling1 and Sampling3 but the virulotypes were not identical (*stx1+ stx2+* and *eae+ stx1+ stx2+*).

### 3.5. Agar Media and PCR-Positive Colonies

The STECB medium gave the highest rate of PCR-positive colonies: 45% of the tested colonies growing on STECB were PCR-positive vs. 3–5% for the other three media. The highest rate of RAMS with PCR-positive colonies was also obtained with the STECB medium (Table 5): 54% vs. 8–12% for the other three media. Similarly, 73% of the 294 PCR-positive colonies and 84% of the 69 RAMS with PCR-positive colonies were obtained with the STECB medium, vs. 2–13% and 7–25% for the other three media, respectively. Of the 52 RAMS with more than one PCR-positive colony, 13 were positive on two agar media, 4 on three agar media, and 2 on all four agar media. Besides the selective properties of ESTe and STECB, there was no correlation between the agar medium and the identified virulotypes.

## 4. Discussion

Using RAMS, <6-month-old dairy calves from three farms in Belgium were identified as healthy carriers not only of (AE-)STEC serotypes but also of EPEC. Although the populations were different (dairy calves and beef cattle), as are the regions in Belgium (Flanders and Wallonia), more calves in farms compared to adult cattle at slaughterhouses harbored one “gang-of-seven” serotype (61% vs. 19%) and more “gang-of-seven“ serotypes (O26, O103, O111, O145, and O157 vs. O26, O103, and O157) were detected during this study than during the study by Thiry and collaborators [17]. Such a higher prevalence of “gang-of-seven” serotypes in young calves has already been reported during different surveys in Australia and New Zealand in dairy, beef, or veal calves compared to adult animals sampled at slaughterhouses [9,10,25].

Nevertheless, the identified serotypes were not evenly distributed in the three farms at the three samplings (Table 4). For instance, the O26 serotype was identified only in FarmB, while the O157 and O103 serotypes were not detected in FarmB and FarmC, respectively. These five serotypes were also not evenly distributed between the pathotypes and virulotypes (Table 3). For instance, the O26 and O103 serotypes were detected only among *eae*+ EPEC and *eae*+ *stx1*+ AE-STEC. In addition, more RAMS harboring AE-STEC isolates (83%) were positive for one of those five serotypes compared to STEC (52%) and EPEC (53%) (Table 3). These latter results are similar to those in diarrheic calves but different from those in healthy cattle in Belgium [13,17]. 

Conversely, the other three serotypes that were looked for in this study (O5, O80, and O118) were not identified, confirming previous findings in Belgium [17,26] and in Europe in general [4] that they are absent, or very rare, in asymptomatic calves and adult cattle. This situation is quite different from the situation in young diarrheic calves that can be infected with O5, O118, and O80, in addition to O26 and O111 AE-STEC and EPEC [12,13,14,16]. Their absence could mean that these O serotypes are present in very low numbers, under the detection level, in healthy calves and adult cattle or that the sampling was not appropriate: recto-anal junction and large intestinal content instead of small intestine in diarrheic calves. 

Of the 69 RAMS (30% of all RAMS) with triplex PCR-positive colonies (Table 1), 36 RAMS (15%) harbored EPEC, 23 RAMS AE-STEC (EHEC) (10%), and 27 RAMS (12%) STEC. The *eae+* EPEC was the most frequent virulotype, while the *eae+ stx1+ stx2+* virulotype was the most frequent among AE-STEC and the *stx1+ stx2+* among STEC. Moreover, 15 RAMS (6%) harbored *E. coli* belonging to more than one virulotype, a situation previously reported in Belgium [27]. These results are to some extent different from those obtained during the study in two slaughterhouses in Belgium [17]. For instance, although the percentage of colon samples harboring triplex PCR-positive colonies was similar (25%), more of the colon samples harbored *E. coli* belonging to different virulotypes (15%). Moreover, though the *eae+* EPEC was the most frequently identified virulotype in both surveys, the most frequent AE-STEC and STEC virulotypes were different: *eae+ stx1+* and *stx2+*.

Differences in the percentage of RAMS positive for the presence of EPEC, AE-STEC, or STEC were observed between farms and between samplings. This percentage was much lower in FarmA (20%) than in Farm B (37%) and FarmC (32%) and at Sampling1 (6%) than at Sampling2 (28%) and Sampling3 (47%). The distributions of pathotypes were also different according to the farms and to the samplings (Table 2). A majority of RAMS were positive for EPEC in FarmA and FarmB and for STEC in FarmC, while a minority of RAMS were positive for AE-STEC in FarmB and for EPEC in FarmC. Not enough RAMS were positive at Sampling1 for a detailed analysis, but the percentage of EPEC-positive RAMS decreased from Sampling2 to Sampling3, while the percentage of STEC-positive RAMS increased and the percentage of AE-STEC-positive remained stable. Several hypotheses may explain such differences between farms and samplings. Were the STEC and EPEC present in such low numbers in FarmA that testing only five colonies per agar medium was not enough? Was FarmA less contaminated with STEC and EPEC? Or were the *stx* genes more unstable in vivo or in vitro in the *E. coli* isolates of FarmA? Similarly, the reason for the increase in RAMS with PCR-confirmed colonies according to the sampling in the three farms (though at different rates) can only be hypothesized, e.g., by the introduction of new asymptomatic carriers and/or by an increase in the shedding of STEC and EPEC in the feces of young or adult animals following some stress. The spread of one (AE-)STEC or EPEC serotype within a farm has been observed as soon as one calf becomes a super-shedder, and the numbers of shedders increase with the number of calves and with their age [11]. Another hypothesis would be the occurrence of EPEC- or AE-STEC-associated diarrhea in some of these young calves. However, this last hypothesis has a very low probability since only one of the 11 diarrheic calves sampled excreted EPEC and no (AE-)STEC. 

Besides their prevalence, the second purpose of this study was to follow the persistence of the different virulotypes and serotypes in the same calves. The results confirm not only that one negative calf can become a shedder a few weeks later, probably after being contaminated by another shedder, but also that EPEC, AE-STEC, and STEC belonging to different virulotypes and serotypes can be excreted at different times by one single calf, illustrating possible multiple contamination events. These results are similar to those obtained by Rice and collaborators [18] who could differentiate between cattle colonized by and cattle transiently shedding O157:H7 *E. coli* using RAMS. Therefore, more than one sampling over a period of several weeks should be recommended when performing a survey in any farm. 

Although fewer healthy dairy calves in those three farms compared to adult cattle in slaughterhouses in Belgium harbored “non-gang-of-seven” serotypes (65% vs. 97%) and although the identification of their actual serotypes will be the purpose of future studies, the question in surveys is the same as previously [17]: how to isolate and identify them. Indeed, so far, the selective methodologies have targeted only the “gang-of-seven” serotypes. The results of this study were obtained using a first enterobacteria enrichment step followed by growth on four (semi-)selective agar media. MC and ES are selective for enterobacteria and coliforms in general, respectively. ESTe and STECB are selective for tellurite-resistant coliforms, including most, if not all, “gang-of-seven” and several “non-gang-of-seven” (AE-)STEC and EPEC, in opposition to the majority of non-STEC, non-EPEC strains [28,29]. This selective property is reflected by the lower number of RAMS with colonies growing on ESTe and STECB (63 and 107 out of 148, respectively) (Table 5). STECB had the highest performance during this study with a similar rate to that in the previous study [17]. While MC and ES also had a similar efficiency in both studies, the situation was highly different for ESTe (7% of RAMS vs. 53% of colon content). The reason for the lower efficiency of ESTe in this study is unknown. 

The choice between a serotype-selective and a non-selective procedure depends on the actual purpose of the study, e.g., whether to study the distribution and circulation of some “gang-of-seven” serotypes or of all EPEC and (AE-)STEC in one farm or one region. This is most important since prevalence and incidence results can differ according to the procedure. For instance, using a serotype-selective procedure on the same RAMS [20], O157 EPEC and (AE-)STEC were detected in FarmA and in FarmC at more sampling times than in this study and O26 EPEC and (AE-)STEC were detected not only in FarmB as in this study but also in FarmA and FarmC. 

The final question is the actual place of the EPEC belonging to the same serotypes as AE-STEC. So far, to the best of our knowledge, no classical genetic method has been able to fully distinguish between true EPEC and AE-STEC having lost the *stx* genes [23,30,31]. Recently, however, we performed a phylogenetic analysis of ca. 50 AE-STEC and EPEC O80:H2 isolated from humans and calves, based on single nucleotide polymorphisms (SNPs), and constructed a maximum likelihood (ML) tree that could distinguish between true EPEC and AE-STEC suspected of having lost the *stx* genes [14]. This may also represent a possibility for analyzing other serotypes containing both AE-STEC and EPEC [1,2,3,4,12,15,19,32,33].

## 5. Conclusions

The results of this survey confirm (i) that, besides suffering diarrhea from AE-STEC and EPEC [12,13,14,15,16], healthy young calves in farms in Belgium can also be asymptomatic carriers of (AE-)STEC and EPEC, at least at the same rate as adult cattle, and (ii) that several (AE-)STEC and EPEC belong to several other O serotypes that are so far unidentified and, thus, are not considered in most surveys.

## Figures and Tables

**Table 1:** Results of the different tests performed on the 233 recto-anal mucosal swabs (RAMS) and isolated colonies from <6-month-old calves in three farms during three sampling times.

Performed Tests	RAMS	Number Positive RAMS (*n* = 69)									
	Farms	FarmA			FarmB			FarmC			Total
	Samplings (S)	S1	S2	S3	S1	S2	S3	S1	S2	S3	
Lauryl sulfate broth triplex PCR		8 (50%) ^1^	8 (33%)	33 (97%)	4 (17%)	23 (88%)	20 (91%)	1 (4%)	19 (76%)	32 (86%)	148 (64%)
Growth on the four agar media ^2^		8 (70) ^3^	8 (131)	33 (495)	4 (42)	23 (326)	20 (270)	1 (14)	19 (294)	32 (504)	148 (2146)
Colony triplex hybridization (CH) ^4^		3 (14) ^5^	3 (4)	13 (50)	3 (14)	13 (59)	12 (58)	1 (5)	8 (34)	21 (100)	77 (338)
Colony triplex PCR ^6^		1 (9) ^5^	1 (2)	13 (42)	2 (10)	12 (51)	12 (52)	1 (3)	8 (34)	19 (91)	69 (294)

^1^ Number PCR-positive RAMS (% of RAMS tested). ^2^ Only the PCR-positive broths were inoculated onto the four agar media. ^3^ Number RAMS with growing colonies (Number colonies picked up). ^4^ Only the growing colonies from the PCR-positive broths were tested via CH. ^5^ Number CH/PCR-positive RAMS (Number positive colonies). ^6^ Only the CH-positive colonies were tested via PCR.

**Table 2:** Pathotypes and virulotypes of the triplex PCR-positive colonies according to the farms and sampling time.

Pathotypes			EPEC	AE-STEC ^1^ (=EHEC)			STEC			PCR-Positive Colonies	
Virulotypes			*eae*	*eae, stx1*	*eae, stx2*	*eae, stx1, stx2*	*stx1*	*stx2*	*stx1, stx2*	Per Farm	Per Sampling
FARMS	A	S1 ^2^	9 (1) ^3^	---	---	---	---	---	---	A: 53 (15)	1: 22 (4)
		S2	--- ^4^	---	2 (1)	---	---	---	---		
		S3	23 (9) ^5^	15 (4)	---	---	---	2 (2)	2 (2)		
	B	S1	9 (1)	---	---	---	---	---	1 (1)	B: 113 (26)	2: 87 (21)
		S2	28 (10)	9 (4)	---	1 (1)	---	13 (3)	---		
		S3	29 (8)	---	1 (1)	5 (2)	5 (4)	12 (3)	---		
	C	S1	---	---	---	---	---	---	3 (1)	C: 128 (28)	3: 185 (44)
		S2	18 (3)	---	---	1 (1)	---	---	15 (4)		
		S3	15 (4)	9 (2)	8 (1)	38 (7)	---	---	21 (8)		
Total virulotypes			131 (36)	33 (10)	11 (3)	45 (11)	5 (4)	27 (8)	42 (16)	294 (69)	
Total pathotypes			131 (36)	89 (23) ^6^			74 (27) ^6^				

^1^ AE-STEC = attaching–effacing Shigatoxigenic *Escherichia coli*. ^2^ S = sampling. ^3^ Number *E. coli* isolates (Number RAMS). ^4^ Number positive colony detected. ^5^ Including one isolate from one diarrheic calf. ^6^ One RAMS harbored *E. coli* belonging to two different virulotypes.

**Table 3:** O somatic serotypes identified among triplex PCR-positive colonies.

O Somatic Serotypes ^1^	EPEC n = 131 (36) ^2^	AE-STEC ^3^ (=EHEC) n = 89 (23)			STEC n = 74 (27)			Total n = 294 (69)
	*eae* n = 131 (36)	*eae,stx1* n = 33 (10)	*eae,stx2* n = 11 (3)	*eae,stx1,stx2* n = 45 (11)	*stx1* n = 5 (4)	*stx2* n = 27 (8)	*stx1,stx2* n = 42 (16)	
O26	4 (3) ^4^	5 (2)	0	0	0	0	0	9 (4)
O103	4 (2)	7 (3)	0	0	0	0	0	11 (4)
O111	5 (4)	2 (2)	0	38 (8)	0	2 (2)	15 (6)	62 (18)
O145	21 (9)	6 (2)	4 (2)	1 (1)	0	4 (2)	7 (3)	43 (16)
O157	4 (1)	0	2 (1)	0	0	0	5 (1)	11 (3)
Total	38 (19)	20 (8)	6 (3)	39 (9)	0/5	6 (4)	27 (10)	136 (42)
	38 (19)	65 (19)			33 (14)			

^1^ O5, O80, O118, O121, and O165 serotypes were not identified. ^2^ Number PCR-positive *Escherichia coli* isolates (Number RAMS with PCR-positive colonies). ^3^ AE-STEC = attaching–effacing Shigatoxigenic *E. coli*. ^4^ Number serotype-positive *E. coli* isolates (Number RAMS).

**Table 4:** Distribution of the O somatic serotypes according to the farms and sampling times in the 42 RAMS with typeable colonies. The O5, O80, O118, O121, and O165 serotypes were not identified.

Sampling/Farm	A	B	C	Total
S1 ^1^	O145 (1) ^2^	O111 (1)	O111 (1)	O111 (2), O145 (1)
S2	O157 (1)	O26 (3), O111 (2), O145 (2)	O111 (3), O145 (2)	O26 (3), O111 (5), O145 (4), O157 (1)
S3	O103 (3), O111 (4) O145 (3)	O26 (1), O103 (1), O145 (4)	O111 (7), O145 (4), O157 (2)	O26 (1), O103 (4), O111 (11), O145 (11), O157 (2)
TOTAL	O103 (3), O111 (4), O145 (4), O157 (1)	O26 (4), O103 (1), O111 (3), O145 (6)	O111 (11), O145 (6), O157 (2)	O26 (4), O103 (4), O111 (18), O145 (16), O157 (3)

^1^ S = sampling. ^2^ O serotype (Number positive RAMS).

**Table 5:** Pathotypes and virulotypes of the triplex PCR-positive colonies according to the agar media.

Pathotypes		EPEC	AE-STEC ^2^ (=EHEC)			STEC			Total PCR-Positive Colonies (%)	Total RAMS with PCR-Positive Colonies (%)
Virulotypes		*eae*	*eae, stx1*	*eae, stx2*	*eae, stx1, stx2*	*stx1*	*stx2*	*stx1, stx2*		
Agar media ^1^	MC	11 (5) ^3^	2 (1)	3 (1)	6 (2)	2 (2)	12 (5)	1 (1)	37 (5%)	16 (11%)
	ES	12 (5)	2 (1)	0	0	3 (2)	14 (6)	3 (3)	33 (5%)	17 (12%)
	ESTe	5 (4)	1 (1)	0	1 (1)	0	0	0	7 (3%)	5 (8%)
	STECB	103 (30) ^4^	28 (10)	8 (3)	38 (11)	0	1 (1)	38 (12)	216 (45%)	58 (54%)
Total virulotypes		131 (36)	33 (10)	11 (3)	45 (11)	5 (4)	27 (8)	42 (16)	294 (14%)	69 (45%)
Total pathotypes		131 (36)	89 (23)			74 (27)				

^1^ MC = McConkey’s; ES = Chromocult Coliform ES; ESTe = Chromocult Coliform ES supplemented with TeK; STECB = supplemented CHROMagar^TM^ STEC base. ^2^ AE-STEC = attaching–effacing Shigatoxigenic *Escherichia coli*. ^3^ Number PCR-positive *E. coli* isolates (Number RAMS with PCR-positive *E. coli* isolates). ^4^ Including one isolate from one diarrheic calf.
